# Transcriptomic Response to Acidosis Reveals Its Contribution to Bone Metastasis in Breast Cancer Cells

**DOI:** 10.3390/cells11030544

**Published:** 2022-02-04

**Authors:** Ana Sayuri Yamagata, Paula Paccielli Freire, Nícolas Jones Villarinho, Ramon Handerson Gomes Teles, Kelliton José Mendonça Francisco, Ruy Gastaldoni Jaeger, Vanessa Morais Freitas

**Affiliations:** 1 Department of Cell and Developmental Biology, Institute of Biomedical Sciences, University of São Paulo, São Paulo 05508-000, Brazil; villarinhonicolas@icb.usp.br (N.J.V.); ramonteles@usp.br (R.H.G.T.); kelliton@usp.br (K.J.M.F.); rgjaeger@usp.br (R.G.J.); vfreitas@usp.br (V.M.F.); 2 Department of Immunology, Institute of Biomedical Sciences, University of São Paulo, São Paulo 05508-000, Brazil; freirepp2@gmail.com

**Keywords:** breast cancer, bone metastasis, acidosis, osteoporosis

## Abstract

Bone is the most common site of metastasis in breast cancer. Metastasis is promoted by acidosis, which is associated with osteoporosis. To investigate how acidosis could promote bone metastasis, we compared differentially expressed genes (DEGs) in MDA-MB-231 cancer cells in acidosis, bone metastasis, and bone metastatic tumors. The DEGs were identified using Biojupies and GEO2R. The expression profiles were assessed with Morpheus. The overlapping DEGs between acidosis and bone metastasis were compared to the bulk of the DEGs in terms of the most important genes and enriched terms using CytoHubba and STRING. The expression of the genes in this overlap filtered by secreted proteins was assessed in the osteoporosis secretome. The analysis revealed that acidosis-associated transcriptomic changes were more similar to bone metastasis than bone metastatic tumors. Extracellular matrix (ECM) organization would be the main biological process shared between acidosis and bone metastasis. The secretome genes upregulated in acidosis, bone metastasis, and osteoporosis-associated mesenchymal stem cells are enriched for ECM organization and angiogenesis. Therefore, acidosis may be more important in the metastatic niche than in the primary tumor. Acidosis may contribute to bone metastasis by promoting ECM organization. Untreated osteoporosis could favor bone metastasis through the increased secretion of ECM organization proteins.

## 1. Introduction

Breast cancer is the most frequent type of cancer in women worldwide. According to Globocan, the age-standardized rate per 100,000 breast cancer patients presented the highest incidence and mortality among all cancer types in 2020 [1,2]. The high mortality rate of breast cancer is often associated with the development of metastasis. Breast cancer is one of the cancer types with the highest prevalence of bone metastasis, with a frequency that ranges from 76 to 100% [3,4]. Complications derived from secondary bone tumors include bone pain, fractures, spinal cord compression, and hypercalcemia of malignancy [5]. It is estimated that 40% of the patients develop bone and visceral metastasis at the same time [6]. On the other hand, patients with bone-only metastasis usually have a better prognosis than visceral-only metastasis [7]. However, compared to breast cancer patients without metastasis, bone metastasis increases the risk of death [8]. In the majority of cases, metastasis is first detected in the bone tissue [9]. Moreover, it has been shown recently that the bone microenvironment prepares cancer cells to disseminate to other organs in a mouse model [10].

It has been suggested that acidosis could contribute to bone metastasis [11], but the molecular pathways underlying this process are still unknown. Consistent with this, Di Pompo et al. (2017) observed that the clone of the human triple-negative breast cancer cell line MDA-MB-231—with a tendency to generate bone metastasis in vivo—acidifies more of the extracellular medium when compared with its parental line. This phenomenon was due to its highly glycolytic metabolism and the increased expression of V-ATPase H+ transporter subunits [12]. When in the bone metastasis microenvironment, acidosis could turn cancer cells more invasive and aggressive [11], induce bone pain [12], and promote bone resorption by osteoclast activation [13].

The bone is essential for the regulation of the acid–base balance [14]. Yet, in chronic acidosis, this regulation is compromised, leading to the loss of bone mass. For instance, extracellular acidosis activates osteoclasts and inhibits osteoblasts [13]. Interestingly, untreated osteoporosis alters the bone microenvironment, possibly fostering the progression of bone metastasis, since the osteoporotic bone is typically acidic [15]. Thus, renal tubular acidosis is a secondary cause of osteoporosis [16]. Breast cancer patients with untreated pre-existing osteoporosis developed bone metastasis sooner than patients without osteoporosis [17].

However, only a few studies have investigated the involvement of acidosis in bone metastasis to this date. To shed light on this subject, transcriptomic changes in MDA-MB-231 in chronic acidosis, bone metastatic tumors, and bone metastasis from mouse xenografts were analyzed. We evaluated if the acidosis-associated transcriptomic changes have more similarity with the changes in the bone metastatic tumor or with the changes in the bone metastasis. Further, we investigated if the shared differentially expressed genes (DEGs) were central components in the different conditions. Finally, we identified the differentially secreted proteins in acidosis and bone metastasis and assessed their levels in the secretome of osteoporosis-associated mesenchymal stem cells.

## 2. Materials and Methods

### 2.1. Datasets

We used RNA-seq data from GSE152345. The triple-negative breast cancer cells MDA-MB-231 were cultured in RPMI-1640 medium (Sigma Aldrich, Catalog No. R1383, Saint Louis, MO, USA) supplemented with 10% fetal bovine serum (Sigma Aldrich, Cat. No. F9665, Saint Louis, MO, USA) and 1% penicillin/streptomycin (Sigma Aldrich, Cat. No. P0781, Saint Louis, MO, USA). The pH of the medium was controlled by addition of NaHCO_3_ and adjustment of the osmolarity with NaCl. The cells were maintained in subculture in 70–90% confluence in pH 7.6 or 6.5. After one month, when equal growth rates were observed, the cells were submitted to RNA isolation and sequencing (RNA-seq) [18].

**Figure 1:**
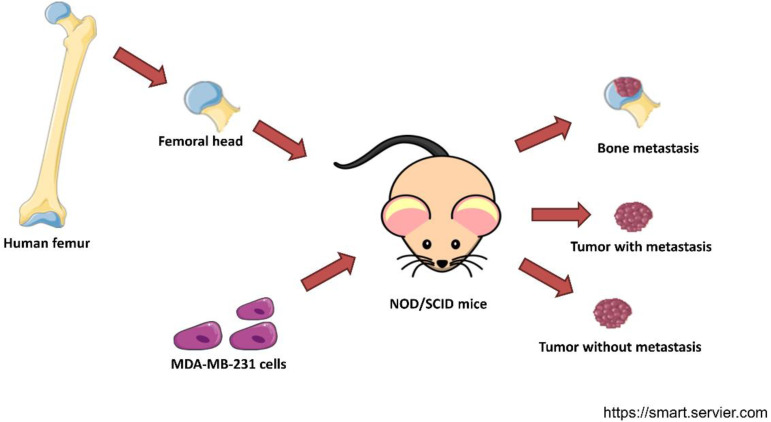
Experimental design of the dataset GSE137842. Trabecular bone fragments from femurs of postmenopausal women were implanted in female NOD/SCID mice. Then, MDA-MB-231 cells were injected into their mammary ducts. cDNA of cancer cells from the primary tumor that developed metastasis, a tumor that did not develop metastasis, and a secondary tumor in the human bone xenograft were subjected to a whole-genome Affymetrix array [19]. This figure was created with templates from https://smart.servier.com/ (accessed on 4 January 2022), a free medical art source licensed under a Creative Common Attribution 3.0 Generic License.

The expression profile of MDA-MB-231 cells in bone metastasis was obtained from GSE137842. Femoral heads from female patients were implanted in female NOD/SCID mice. After four weeks, MDA-MB-231-luc2-TdTomato cells were orthotopically injected (Figure 1). When the tumors were 1cm^3^ or the body weight was ≥10% lower, the mice were euthanized. MDA-MB-231-luc2-TdTomato from the primary tumors that caused metastasis, primary tumors that did not cause metastasis, and metastasis in the human bone were extracted from whole blood. TdTomato-positive tumor cells were isolated by flow cytometry sorting. Total RNA was extracted and converted to cDNA. The samples were analyzed in a whole-genome Affymetrix array [19].

We also analyzed the transcriptomic data of mesenchymal stem cells from femoral heads of women with or without osteoporosis from the dataset GSE35959. The age of the two groups passed the Shapiro–Wilk test for normality, and no difference between them was detected in the *t*-test with Welch’s correction (Table 1). Biopsies were collected by low-energy fracture from patients undergoing hip arthroplasty. The mesenchymal stem cells were extracted following the originally cited protocol [20] and isolated by adherence to a culture plate. The cells were cultured in DMEM/Ham’s F-12 (1∶1) medium supplemented with 10% fetal calf serum, 1 U/mL penicillin, 100 µg/mL streptomycin, and 50 µg/mL L-ascorbic acid 2-phosphate (Sigma Aldrich GmbH, Schnelldorf, Germany) [20]. At 1st or 2nd passage, total RNA was isolated and submitted to a microarray analysis [20].

### 2.2. Preliminary Analysis of RNAseq Dataset

The raw data of GSE152345 was downloaded from the Gene Expression Omnibus (GEO) database (https://www.ncbi.nlm.nih.gov/geo/, accessed on 10 January 2022). The gene identifiers (Ensembl IDs) were then converted to their gene symbol using the ENSEMBL Gene ID to Gene Symbol Converter (https://www.biotools.fr/human/ensembl_symbol_converter, accessed on 14 May 2021). The data of MDA-MB-231 in pH 7.6 and pH 6.5 were selected and uploaded to BioJupies [21]. The differential expression table was obtained in BioJupies after selecting which sample to compare.

### 2.3. Preliminary Analysis of Affymetrix Array Dataset

The dataset of MDA-MB-231 in bone metastasis (GSE137842) and the dataset of mesenchymal stem cells in osteoporosis were first analyzed with GEO2R. Using this tool, the data was normalized in terms of log2(fold change) (logFC) The Benjamini & Hochberg correction for multiple testing and limma precision weights were applied.

### 2.4. Identification of Differentially Expressed Genes (DEGs)

Genes with a *p*-value higher than 0.05 were filtered out. If log(FC) > 1.5, the gene was considered upregulated. If logFC < −1.5, it was considered downregulated. Venn diagrams were created using the webtool from Bioinformatics & Evolutionary Genomics (http://bioinformatics.psb.ugent.be/webtools/Venn/, accessed on 9 January 2022). The overlap of DEGs was considered more adequate than just the enrichment terms to identify potential molecular mechanisms because enrichment terms can comprise a large number of genes and a broad classification of a biological process.

### 2.5. Heatmaps

Heatmaps of gene expression data were created using Morpheus [22]. The color scheme was relative to each row. The lines and rows were organized by hierarchical clustering by Euclidean distance of average.

### 2.6. Enrichment Analysis

The lists of DEGs were submitted to enrichment analysis to identify enriched Gene Ontology terms. The Biological Processes were identified through the STRING app [23] for Cytoscape [24]. The enriched terms were ranked by *p*-value. This app can be used for transcriptome data as well [23].

### 2.7. Interactome Analysis

The protein–protein interaction (PPI) networks were retrieved with the STRING app for Cytoscape [23]. Information regarding text mining, experiments, databases, co-expression, neighborhood, gene fusion, and co-occurrence was considered in the elaboration of the networks. The aliases for gene names were verified in genecards (https://www.genecards.org/, accessed on 4 August 2021) to solve ambiguity before submission. A confidence score cutoff of 0.4 was employed and no interactors were added. The nodes without any connection were excluded from the network to likely evidence genes cohesively acting together in certain biological processes rather than merely random genes with distinguishable expression between groups. The nodes were ranked with the MCC ranking method in the CytoHubba app for Cytoscape [25]. This network analysis enabled us to identify the most important nodes, and thus the potentially most likely mediators of the processes involved.

### 2.8. Secretome Prediction from Transcriptome

The DEGs in osteoporosis vs. control were filtered for predicted secreted proteins, listed in The Human Protein Atlas (https://www.proteinatlas.org/search/protein_class%3APredicted+secreted+proteins, accessed on 1 January 2022) [26]. The list comprises proteins predicted by all the methods used: MDSEC, signal, Phobius, and STOPTOPUS. Further, the proteins were filtered for the overlap of upregulated proteins in acidosis and bone metastasis. In this way, we found some proteins that could be highly secreted in osteoporosis, and added to the proteins secreted by cancer cells in acidosis and bone metastasis.

## 3. Results

**Figure 2:**
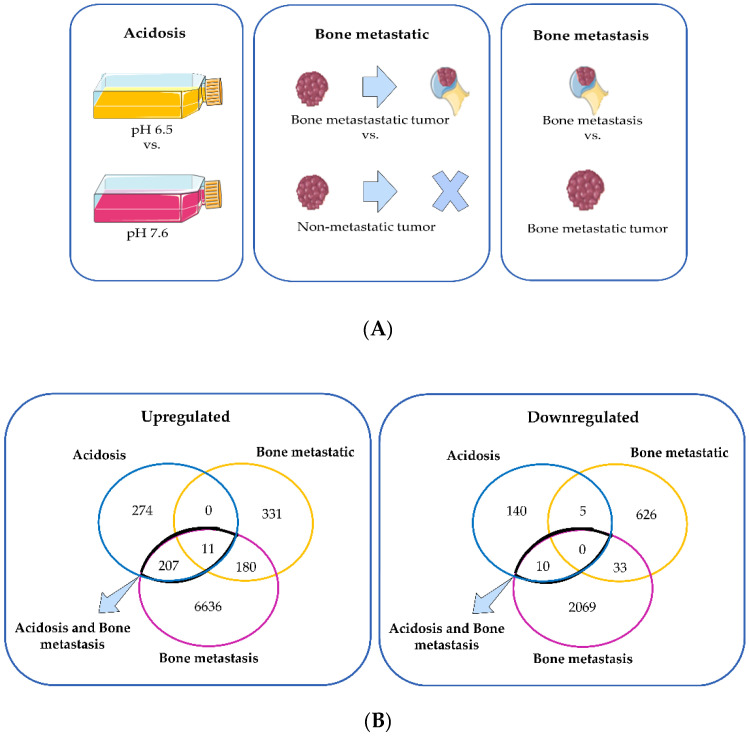
(**A**) Schematic representation of the comparisons made between the transcriptome of MDA-MB-231 in different conditions: long-term culture in pH 6.5 relative to long-term culture in pH 7.6 (acidosis); bone metastatic primary tumor relative to non-metastatic primary tumor (bone metastatic); bone metastasis relative to bone metastatic primary tumor (bone metastasis). (**B**) Venn diagram of the upregulated and downregulated genes of the three comparisons: “acidosis”, “bone metastatic”, and “bone metastasis”. The Figure 2A was created with templates from https://smart.servier.com/ (accessed on 4 January 2022), a free medical art source licensed under a Creative Common Attribution 3.0 Generic License.

**Figure 3:**
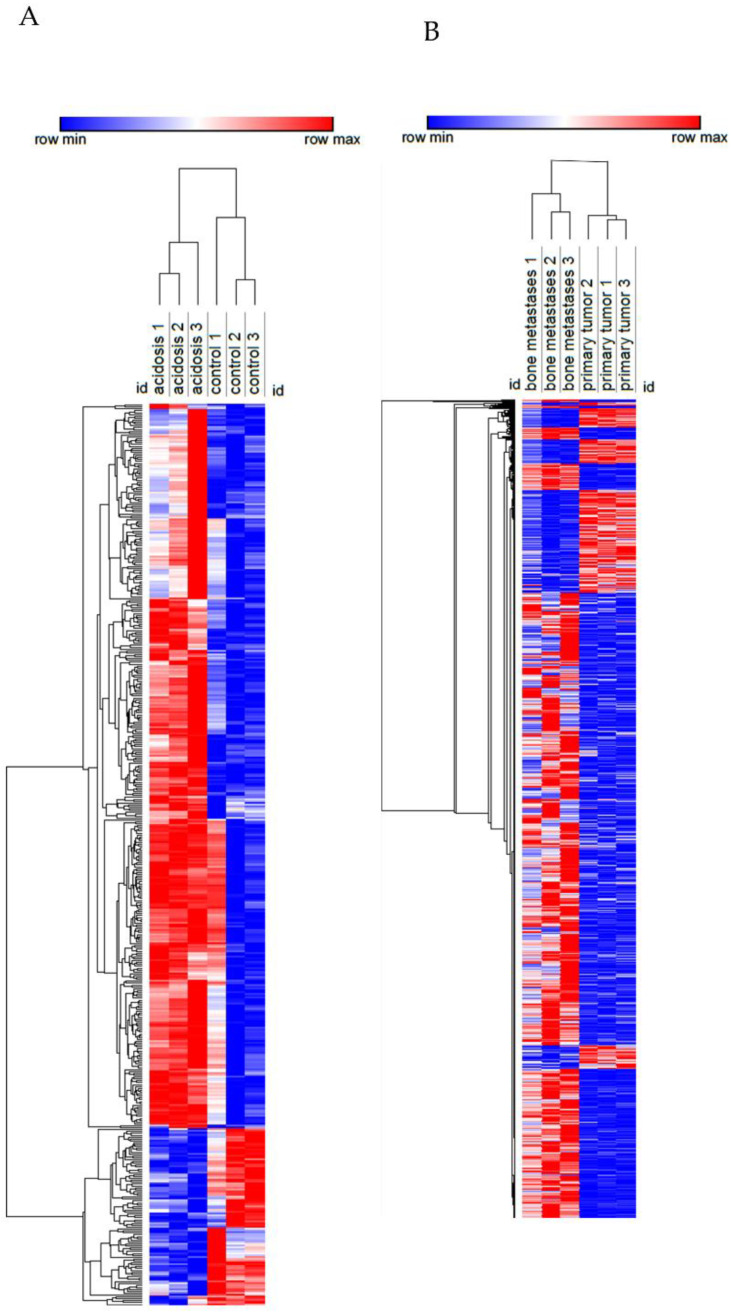
Heatmap of the transcriptome of MDA-MB-231 in chronic acidosis (**A**) and in MDA-MB-231 from bone metastasis in a mouse xenograft model (**B**). Rows (differentially expressed genes) and columns (samples) were clustered hierarchically by Euclidian distance. Red and blue correspond to maximum and minimum values relative to row average.

To characterize the changes induced by acidosis in triple-negative breast cancer, we used a dataset of MDA-MB-231 cells adapted at pH 6.5 or cultured at pH 7.6. To compare these changes of acidosis to the ones induced by the bone metastatic microenvironment and the bone metastasis microenvironment, we used a dataset of MDA-MB-231 from the primary tumor and secondary tumors in human bone xenografted into mice. The differentially expressed genes (DEGs) in chronic acidosis were called “Acidosis” and may be important for adaptation to extracellular acidosis. The DEGs in the bone metastatic tumor, compared to the non-metastatic tumors, were called “Bone metastatic” and may regulate tropism to the bone. “Bone metastasis” refers to the DEGs in bone metastasis and may be important for the establishment and colonization of cancer cells in the bone secondary tumor microenvironment (Figure 2A). Interestingly, “Acidosis” had more upregulated and downregulated genes in common with “Bone metastasis” than with “Bone metastatic” (Figure 2B, Table 2). Therefore, we focused on the relationship between acidosis and the bone metastasis microenvironment. Figure 3 shows the relative intensity of gene expression in each row, wherein each column is a sample. The transcriptomic alterations associated with chronic acidosis and the alterations associated with bone metastasis are similar among replicates of the same group. In both comparisons, there were abundantly more upregulated than downregulated genes.

Next, we evaluated the expression of genes that are important for bone metastasis in chronic acidosis (Table 3). Among IL-6, Wnt ligands, HIF-1α, cathepsin K, and cathepsin L, only IL-6, WNT2B, WNT3, and WNT11 had *p*-value < 0.05. Of those, only WNT3 had logFC < 1.5.

Conversely, genes linked to adaptation to acidosis were analyzed in bone metastasis and bone metastatic tumors (Table 4). The increase in the expression of ATPase H+ transporter subunits was one of the changes we expected to see in cancer cells from bone metastasis, as they are biomarkers of adaptation to acidosis [12]. The cancer cells from bone metastasis had a higher expression of ATPase H+ transporter subunits, while these genes were not upregulated in the cells from bone metastatic tumors.

**Figure 4:**
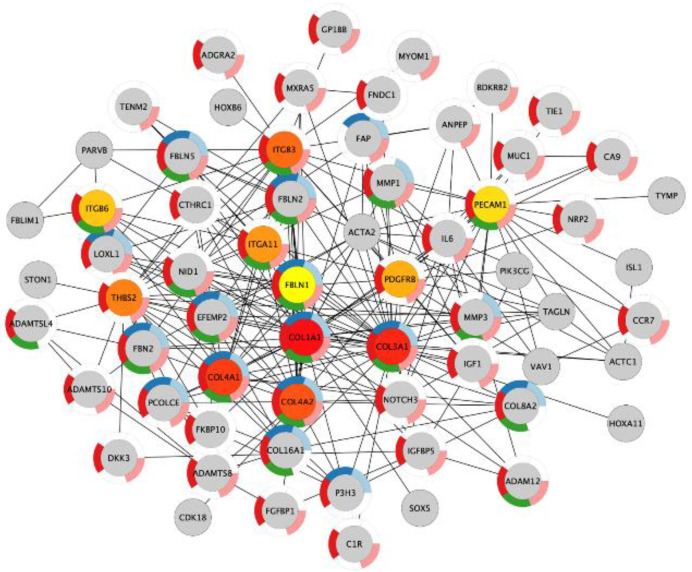
The ten most important nodes in the protein–protein interaction network of the genes with increased expression in chronic acidosis in MDA-MB-231. The intensity of the red color of the nodes corresponds to their rank within the network. The color of the donut-shaped outliner of each node is related to the enriched Gene Ontology Biological Process terms of the Table 5.

**Figure 5:**
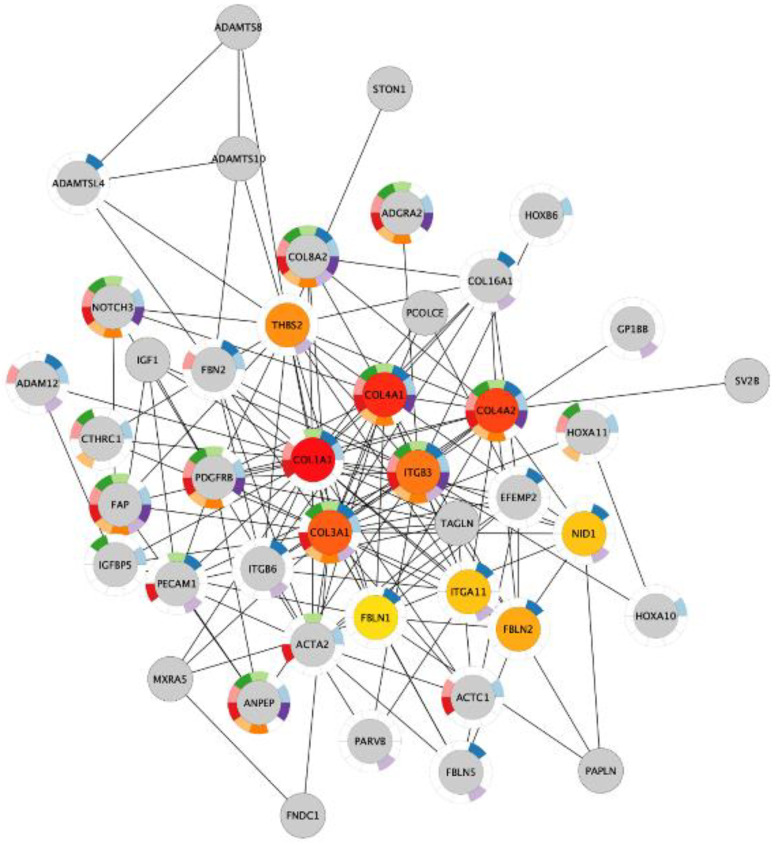
The ten most important nodes in the protein–protein interaction network of the genes in the intersection between the upregulated genes in chronic acidosis and the upregulated genes in bone metastasis in MDA-MB-231 xenograft. The intensity of the red color of the nodes corresponds to their rank within the network. The color of the donut-shaped outliner of each node is related to the enriched Gene Ontology Biological Process terms of the Table 6.

Next, we investigated if the shared upregulated genes in acidosis and bone metastasis belonged to the most important changes in acidosis. The overlap “Acidosis and Bone metastasis” was compared to “Acidosis”. The top five enrichment terms in “Acidosis” (Table 5) were also among the six more enriched terms in “Acidosis and Bone metastasis” (Table 6). These findings indicate that the adaptation of cancer cells to the bone microenvironment would favor fitness in acidic conditions by remodeling the extracellular matrix and enhancing angiogenesis. The bone marrow is more vulnerable to oxygen fluctuations [33], so angiogenesis could be a strategy to survive in this microenvironment. Additionally, the top seven nodes of the “Acidosis” network (Figure 4) were present among the top ten nodes of “Acidosis and Bone metastasis” (Figure 5): COL1A1, COL3A1, COL4A1, COL4A2, ITGB3, THBS2, and ITGA11. In other words, the main genes involved in adaptation to acidosis are included in the transcriptomic changes in MDA-MB-231 bone metastasis.

We also investigated if the upregulated genes in both acidosis and bone metastasis were among the most important transcriptomic changes in bone metastasis. Four hundred and twenty-eight Gene Ontology Biological Process terms were identified from the enrichment analysis of the upregulated genes in bone metastasis. Interestingly, the 10 most enriched terms in the upregulated genes in acidosis and bone metastasis were found among the 89 terms with the lowest *p*-value (Table 7).

This data suggests that the most prominent contribution of acidosis to the colonization of bone metastasis may be the organization of the extracellular matrix, mainly by the expression of collagen.

**Figure 6:**
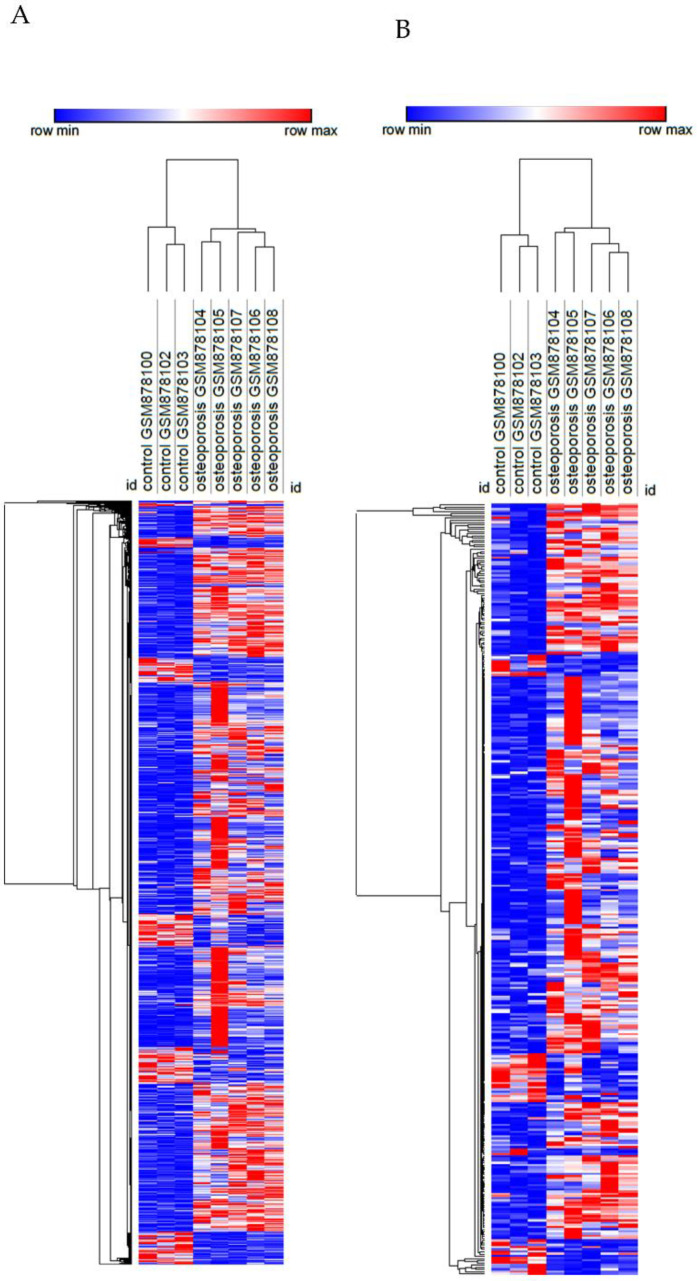
Heatmap of the transcriptome (**A**) and transcriptome-based secretome (**B**) of mesenchymal stem cells from femoral heads of female patients with or without osteoporosis. Rows (differentially expressed genes) and columns (samples) were clustered hierarchically by Euclidian distance. Red and blue correspond to maximum and minimum values relative to row average.

**Figure 7:**
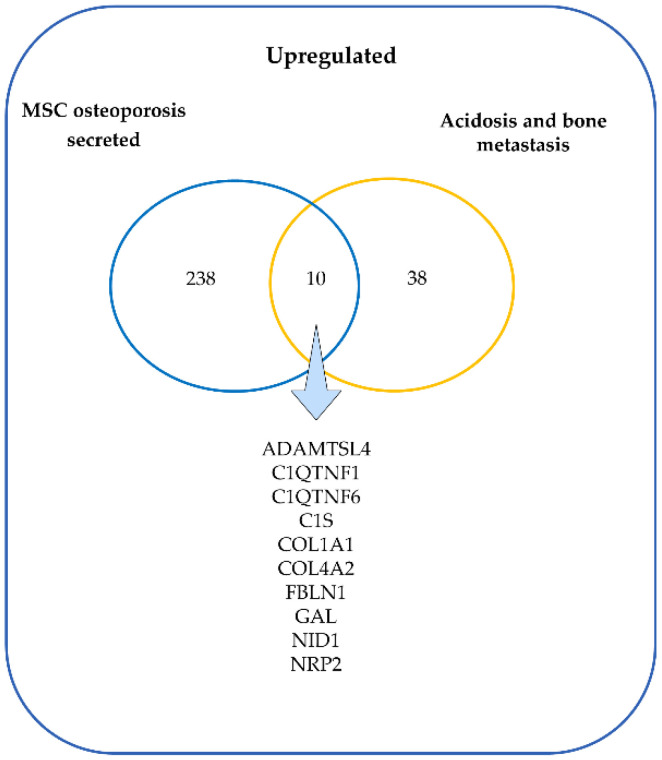
Venn diagram of the upregulated genes in the secretome of mesenchymal stem cells (MSC) from osteoporosis patients and the upregulated genes in MDA-MB-231 in acidosis and bone metastasis.

Finally, we identified which upregulated genes in acidosis and bone metastasis with predicted secretion were also upregulated in the osteoporosis secretome. The two groups of samples—osteoporosis and control—were distinguishable by DEGs and by DEGs of the transcriptome-based secretome (Figure 6). Overall, there were more upregulated than downregulated genes. Ten overlapping genes were found: ADAMTSL4, C1QTNF1, C1QTNF6, C1S, COL1A1, COL4A2, FBLN1, GAL, NID1, and NRP2 (Figure 7). Interestingly, all of the ten most enriched terms in acidosis and bone metastasis were also enriched in the secretome of mesenchymal stem cells (MSC) in osteoporosis (Table 8).

## 4. Discussion

Cancer cells that thrive in the adverse conditions of the tumor microenvironment undergo numerous modifications [34]. For instance, cancer cells in the glandular lumen of breast tumors translocate more LAMP2 to the outer surface of the membrane as an adaptative mechanism to acidosis due to low vascularization [35,36]. Additionally, the circulating cancer cells (CTCs) endure various selective pressures until they reach a pre-metastatic niche in a process well-described elsewhere [37,38,39,40]. One of the first challenges CTCs face is detachment from the extracellular matrix. Breast cancer cells adapted to acidosis acquire resistance to anoikis by the increased production of fibrillar collagen, including COL4A2, therefore actively depositing extracellular matrix [41]. Prior to CTC detachment, the pre-metastatic niche is already undergoing modifications that favor their seeding. In breast cancer, this process of pre-metastatic niche formation starts concomitantly with the primary tumor growth, in which the brain, liver, lungs, and bones are the most common organs to receive breast cancer cells [3]. For instance, IL-6 and TGF-β secreted by bone marrow mesenchymal stem cells facilitate metastasis [42,43]. However, the mechanisms through which acidosis would foster breast cancer bone metastasis have not been investigated yet. This exploratory study intended to direct future research on this subject.

The alterations in the expression profile of the breast cancer cells MDA-MB-231 induced by chronic acidosis were more similar to the changes in these cells in bone metastasis than in bone metastatic tumors (Figure 2B). This suggests that the cancer cells in bone metastasis could be more adapted to acidosis than the cancer cells in the bone metastatic tumor. Alternatively, the genes upregulated in response to acidosis might favor the outgrowth of bone metastasis rather than the spread of primary tumor metastatic cells. Interestingly, it has been proposed that the bone secondary tumor microenvironment may prepare breast cancer cells to become more competent to metastasize in visceral organs as they thrive in adverse conditions, such as oxidative stress [44]. This could explain the better prognosis of breast cancer patients with metastasis restricted to the bone compared to bone and visceral metastases simultaneously or with brain, liver, or lung solely [7]. In line with this rationale, ATPase H+ transporter subunits were more expressed in bone metastasis compared to the primary tumor (Table 4). V-ATPase subunits can be located in the cytoplasmic membrane, activate proteases, and degrade the extracellular matrix [45]. They are important for the progression of bone metastasis [46,47]. An isoform of the C subunit was identified to be overexpressed in 34% of human breast cancer cases and was associated with poor survival, primary tumor growth, and bone metastasis formation [48]. Other isoforms are also related to a more aggressive cancer phenotype, as V1B1 and V1G1 were found with high expression levels in a subclone of MDA-MB-231 breast cancer cells with tropism to bone [12]. These findings corroborate that cancer cells in bone metastasis must be more adapted to the acidic milieu. Ion/protons pumps are expressed in both tumor and tumor-associated normal cells, in which the vacuolar H^+^-ATPase (V-ATPase) is defined as the most important for bone metastasis progression [46]. It is a family of pumps, formed by two domains and different subunits, located on the lysosomal membrane that acidifies the intralysosomal space; in osteoclasts it can also be found on the cytoplasmic membrane, activating proteases, and degrading the extracellular matrix [45].

Additionally, the increase in gene expression of IL-6 and Wnt ligands, such as WNT2B, 3 and 11, associated with acidosis (Table 3) could contribute to bone metastasis. IL-6 secreted continuously from cancer cells in the bone marrow and promotes osteoclasts differentiation [27]; and Wnt signaling in breast cancer stem cells enables colonization in the bone [28].

The genes that were upregulated in both acidosis and bone metastasis were mainly related to the organization of the extracellular matrix and angiogenesis. This overlap corresponds to the most important biological processes involved in the adaptation to acidosis and in bone metastasis separately. An enrichment of up- and downregulated genes related to extracellular matrix organization and angiogenesis was found in both acid-adapted cancer cells and tumor vs. control tissue from patients [18]. However, for the first time, we observed that these biological processes can be the most prominent ones involved in the contribution of acidosis to bone metastasis. We identified the main genes probably orchestrating adaptation of MDA-MB-231 cells in chronic acidosis that may contribute to bone metastasis: COL1A1, COL4A1, COL4A2, COL3A1, ITGB3, THBS2, and ITGA11 (Figure 5). ITGB3, ITGA11, and THBS2, which all encode integrin subunits beta 3, alpha 11, and Thrombospondin 2, respectively. Mutations in these proteins are associated with gastric [49], lung [50], and breast cancer [51].

Thrombospondins are secreted glycoproteins involved in various cellular events, such as angiogenesis and inflammatory response [52]. Thrombospondin 2 (THBS2) stimulates osteoclastogenesis in a receptor activator of nuclear factor kappa-Β ligand (RANKL) dependent way, promoting osteolytic lesions in lung cancer-derived bone metastasis [53]. The interaction of RANKL with RANK is widely known to activate signaling events for the differentiation of osteoclasts [54]. On the other hand, THBS2 facilitates the differentiation of osteoblasts [55] and helps build consistency in the connective tissue, including the bone matrix [56]. THBS2 maturates COL1A1 by an increase in the expression of lysyl oxidase (LOX) [57] and incorporates it into the ECM [55]. Due to its role in the ECM, THBS2 augments cancer cell migration [58]. In fact, in tumor extracellular matrix proteomic analysis from breast tumors, THBS2, followed by FBLN1 and COL1A1, have the highest correlation with collagen fiber alignment [59], an ECM trait predictive of shorter disease-free survival [60].

Integrin β3 intermediates the adhesion of cancer cells to the bone matrix proteins, including collagen [61]. Additionally, the heterodimer αvβ3 in tumor cells is responsible for invasion of the bone and the recruitment of osteoclasts [62]. In a study evaluating the integrin β3 inhibition, anti-proliferative and anti-migratory effects were observed in MDA-MB-231 cells, as well as complete remission of osteolytic lesions in a nude rat model of bone metastasis [63]. There are few studies involving integrin α11 and bone tissue [64]. ITGA11 content in stroma contributes to the tumor growth and aggressiveness in both MDA-MB-231 xenograft models [65] and breast cancer patients [66]. Stromal ITGA11 is also associated with the triple-negative phenotype [66].

Collagen corresponds to 90% of the bone organic extracellular matrix before non-fibrous proteins (10%) [67]. The extracellular matrix modification is a hallmark of cancer invasion [68]. Collagen density in the mammary stroma was causally linked to tumor growth and lung metastasis [69]. Collagen, the most abundant matrix polymer, increases tumor tissue stiffness, regulates tumor immunity, and promotes metastasis [70]. ECM collagen fibers, disposed radially from the tumor core, increase stiffness in the periphery [71]. This stiff ECM architecture increases cell migration, providing a leading “highway” and promoting cellular contraction, and it obstructs drug delivery due to the elevated interstitial fluid pressure [72]. It has been shown recently that conditioning to ECM stiffness in breast cancer favors bone metastasis [73,74]. Triple-negative breast cancer shows increased deposition of collagen and enhanced invasion of cancer-associated fibroblasts [75,76]. Collagen was simultaneously increased in acidosis and bone metastasis, and mainly relates to the extracellular matrix, cell adhesion, and angiogenesis (Figure 5, Table 6). Extracellular matrix organization must play a crucial role in the contribution of acidosis to bone metastasis development, since it was one of the most enriched biological process terms in the genes increased in “Acidosis and Bone metastasis” (Table 6).

COL4A2 was found among the top ten important genes in acidosis and the “Acidosis and Bone metastasis” overlap (Figure 4 and Figure 5). NOTCH3 was also included, interacting with this gene in both cases. NOTCH3 increases the expression of COL4A2, which makes cancer cells more resistant to anoikis [77]. Moreover, COL4A2 enhances MDA-MB-231 migration and proliferation [78]. Accordingly, acid-adapted MCF7 also has increased mRNA levels of COL4A2 [41]. The higher levels of circulating collagen IV in breast cancer patients with metastasis than in patients without metastasis illustrates the role of COL4A2 in the development of metastasis [79].

The comparison between the upregulated genes in both acidosis and bone metastasis and the secretome of bone marrow MSC from osteoporosis patients revealed ten genes that could mediate the involvement of osteoporosis in bone metastasis. Among these genes were COL1A1, COL4A2, NID1, FBLN1, and NRP2. NRP2 inhibits osteoclast activity and promotes the tumor burden in bone with mixed lesions [80]. Increased expression of COL1A1 and COL1A2 are considered to influence tumor invasion and progression and are reported in several types of cancer, such as gastric [49], colorectal [81], and breast cancer [82,83]. COL1A1 is regulated by MRTF-A in the Wnt/β-catenin-induction, which integrates signals from various pathways to control the Type I collagen synthesis, reciprocally stimulating the signal transduction in cancer cells—besides promoting metastasis and proliferation [83]. The anchoring of tumor cells mediated by integrins activates Wnt-dependent intracellular signaling pathways, ensuring the survival of tumor cells colonizing the bone tissue [67]. The expression of COL1A1 promotes migration of MDA-MB-231 in vitro [84].

Confirmative experiments for validation were not possible due to the complexity of the experimental design of the dataset used. Given that we focused on the bone as a secondary tumor microenvironment, there were no large datasets of transcriptome from bulk tissue of breast cancer bone metastasis to compare the findings. However, the use of a well-known human triple-negative breast cancer cell line in culture and the same cell line isolated from the primary and secondary tumors enabled an accurate comparison between the differentially expressed genes in acidosis, bone metastatic tumor, and bone metastases without contamination of other cell types. Besides, the xenografted human bone was a secondary tumor microenvironment much more representative than the mouse bone.

Our results indicate that the main transcriptomic changes in triple-negative breast cancer cells induced by chronic acidosis may contribute to the colonization of breast cancer cells in the bone. The cancer cells would become more competent to survive, proliferate, and spread in this environment through organization of the extracellular matrix, angiogenesis, cell adhesion, and migration. Furthermore, the generally acidic osteoporotic bone in untreated osteoporosis could support bone metastasis with the secretion of proteins such as COL1A1, COL4A2, NID1, FBLN1, and NRP2. For the first time, the association between adaptation to chronic acidosis, alterations in the expression profile in bone metastasis, and the secretome of MSC in osteoporosis was explored.

## Figures and Tables

**Table 1:** Age of the female participants analyzed from the dataset GSE35959.

	Control	Osteoporosis	*p*-Value
n	3	5	
Age (years)	82.67 (±3.18)	86.20 (±2.634)	0.4336

Values expressed as mean ± standard error of the mean.

**Table 2:** Percentage of differentially expressed genes in acidosis that overlapped with bone metastatic tumor or bone metastasis.

	% of the Upregulated Genes in Acidosis	% of the Downregulated Genes in Acidosis
Bone metastatic	2.24	3.22
Bone metastasis	44.3	6.45

**Table 3:** Genes with known importance to bone metastasis colonization assessed in the transcriptome of MDA-MB-231 in chronic acidosis. * *p*-value < 0.05.

Acidosis vs. Control					
Gene Symbol	Gene Title	logFC	*p*-Value	adj. *p*-Value	Reference
IL6	Interleukin 6	3.212	0.04 *	0.265	[27]
WNT2B	Wnt Family Member 2B	1.959	0.015 *	0.193	[28]
WNT3	Wnt Family Member 3	0.583	0.013 *	0.187	[28]
WNT5A	Wnt Family Member 5A	1.001	0.193	0.482	[28]
WNT5B	Wnt Family Member 5B	−0.073	0.832	0.925	[28]
WNT7B	Wnt Family Member 7B	0.340	0.520	0.750	[28]
WNT9A	Wnt Family Member 9A	−0.708	0.078	0.335	[28]
WNT10B	Wnt Family Member 10B	1.071	0.105	0.380	[28]
WNT11	Wnt Family Member 11	4.035	0.001 *	0.094	[28]
HIF1A	Hypoxia Inducible Factor 1 Subunit Alpha	0.051	0.820	0.919	[29]
CTSK	Cathepsin K	0.475	0.236	0.526	[30,31]
CTSL	Cathepsin L	0.594	0.078	0.335	[32]

**Table 4:** ATPase H+ transporter subunits in MDA-MB-231 cells from bone metastasis relative to its primary tumor and bone metastatic tumor relative to non-metastatic tumor in a mouse xenograft model. * *p*-value < 0.05.

**Bone Metastases vs. Metastatic Tumor**				
**Gene Symbol**	**Gene Title**	**logFC**	***p*-Value**	**adj. *p*-Value**
ATP6AP1L	ATPase H+ transporting accessory protein 1 like	2.565	4.13 × 10^−4^ *	3.28 × 10^−3^
ATP6AP1L	ATPase H+ transporting accessory protein 1 like	3.589	8.89 × 10^−5^ *	1.06 × 10^−3^
ATP6V0D2	ATPase H+ transporting V0 subunit d2	1.706	2.66 × 10^−4^ *	2.35 × 10^−3^
ATP6V0D2	ATPase H+ transporting V0 subunit d2	2.2	1.22 × 10^−5^ *	2.61 × 10^−4^
ATP6V0D2	ATPase H+ transporting V0 subunit d2	3.285	2.40 × 10^−3^ *	1.23 × 10^−2^
ATP6V1C1	ATPase H+ transporting V1 subunit C1	3.086	6.33 × 10^−3^ *	2.56 × 10^−2^
ATP6V1H	ATPase H+ transporting V1 subunit H	1.580	1.30 × 10^−3^ *	7.77 × 10^−3^
**Bone metastatic tumor vs. non-metastatic tumor**				
**Gene symbol**	**Gene title**	**logFC**	***p*-value**	**adj. *p*-value**
ATP6AP1L	ATPase H+ transporting accessory protein 1 like	−1.192	0.096	0.958
ATP6AP1L	ATPase H+ transporting accessory protein 1 like	0.289	0.638	0.98
ATP6V0D2	ATPase H+ transporting V0 subunit d2	0.281	0.417	0.969
ATP6V0D2	ATPase H+ transporting V0 subunit d2	−0.037	0.905	0.996
ATP6V0D2	ATPase H+ transporting V0 subunit d2	−0.549	0.611	0.979
ATP6V1C1	ATPase H+ transporting V1 subunit C1	0.075	0.787	0.989
ATP6V1H	ATPase H+ transporting V1 subunit H	−0.036	0.874	0.994

**Table 5:** The Five Enriched Gene Ontology Biological Process terms with the lowest *p*-value in the upregulated genes in chronic acidosis in MDA-MB-231. The associated colors indicate the nodes respective to each term in the Figure 4.

	Acidosis		Nodes: 374	Edges: 502		
Color	GO Term Name	Description	FDR	*p* Value	Genes	Background Genes
	GO.0030198	extracellular matrix organization	9.14 × 10^−6^	2.21 × 10^−9^	25	296
	GO.0001568	blood vessel development	8.78 × 10^−5^	6.36 × 10^−8^	29	464
	GO.0009653	anatomical structure morphogenesis	2.10 × 10^−4^	3.11 × 10^−7^	71	1992
	GO.0048646	anatomical structure formation involved in morphogenesis	7.20 × 10^−4^	1.21 × 10^−6^	38	831
	GO.0072359	circulatory system development	8.30 × 10^−4^	1.59 × 10^−6^	37	807

**Table 6:** The Ten Enriched Gene Ontology Biological Process terms with the lowest *p*-value in the intersection between the upregulated genes in chronic acidosis and the upregulated genes in bone metastasis of MDA-MB-231 mouse xenograft. The associated colors indicate the nodes respective to each term in the Figure 5.

	Acidosis and Bone Metastasis					
			Nodes: 205	Edges: 263		
Color	GO Term Name	Description	FDR	*p* Value	Genes	Background Genes
	GO.0009653	anatomical structure morphogenesis	1.91 × 10^−8^	6.22 × 10^−12^	56	1992
	GO.0030198	extracellular matrix organization	2.67 × 10^−8^	1.74 × 10^−11^	21	296
	GO.0001568	blood vessel development	5.37 × 10^−8^	5.25 × 10^−11^	25	464
	GO.0035295	tube development	2.15 × 10^−7^	4.90 × 10^−10^	31	793
	GO.0048646	anatomical structure formation involved in morphogenesis	5.57 × 10^−7^	1.45 × 10^−9^	31	831
	GO.0072359	circulatory system development	1.02 × 10^−6^	2.98 × 10^−9^	30	807
	GO.0035239	tube morphogenesis	1.02 × 10^−6^	3.01 × 10^−9^	26	615
	GO.0048514	blood vessel morphogenesis	2.12 × 10^−6^	7.59 × 10^−9^	20	381
	GO.0007155	cell adhesion	7.48 × 10^−6^	2.93 × 10^−8^	29	843
	GO.0001525	Angiogenesis	7.67 × 10^−6^	3.25 × 10^−8^	17	297

**Table 7:** The ten most enriched Gene Ontology Biological Process terms in acidosis and bone metastasis observed in the enrichment analysis of the upregulated genes in bone metastasis of MDA-MB-231 mouse xenograft. The terms were ranked from the lowest to the highest *p*-value.

	Bone Metastasis					
			Nodes: 5676	Edges: 88,598		
Rank	GO Term Name	Description	FDR	*p*-Value	Genes	Background Genes
3	GO:0007155	Cell adhesion	2.71 × 10^−24^	4.64 × 10^−28^	495	925
9	GO:0009653	Anatomical structure morphogenesis	5.26 × 10^−20^	2.71 × 10^−23^	913	2165
38	GO:0072359	Circulatory system development	1.90 × 10^−10^	4.13 × 10^−13^	392	872
39	GO:0048646	Anatomical structure formation involved in morphogenesis	2.56 × 10^−10^	5.70 × 10^−13^	395	883
50	GO:0035295	Tube development	7.10 × 10^−9^	2.03 × 10^−11^	373	851
57	GO:0035239	Tube morphogenesis	1.35 × 10^−8^	4.39 × 10^−11^	301	656
69	GO:0030198	Extracellular matrix organization	5.49 × 10^−8^	2.20 × 10^−10^	178	338
74	GO:0001568	Blood vessel development	8.97 × 10^−8^	3.89 × 10^−10^	238	500
81	GO:0048514	Blood vessel morphogenesis	4.72 × 10^−7^	2.24 × 10^−9^	200	410
89	GO:0001525	Angiogenesis	3.41 × 10^−6^	1.77 × 10^−8^	159	315

**Table 8:** The ten most enriched Gene Ontology Biological Process terms in acidosis and bone metastasis observed in the enrichment analysis of the upregulated genes in the transcriptome-based secretome of mesenchymal stem cells from bone of osteoporosis patients. The terms were ranked from the lowest to the highest *p*-value.

	Osteoporosis Secretome					
			Nodes: 280	Edges: 1447		
Rank	GO Term Name	Description	FDR	*p*-Value	Genes	Background Genes
1	GO:0030198	Extracellular matrix organization	2.53 × 10^−18^	1.44 × 10^−22^	39	338
11	GO:0001568	Tube morphogenesis	3.75 × 10^−14^	2.36 × 10^−18^	46	656
12	GO:0006952	Blood vessel morphogenesis	2.21 × 10^−17^	3.67 × 10^−18^	37	410
13	GO:0048583	Angiogenesis	7.54 × 10^−17^	4.67 × 10^−18^	33	315
15	GO:0009605	Tube development	1.34 × 10^−16^	1.39 × 10^−17^	51	851
18	GO:2000145	Blood vessel development	1.34 × 10^−16^	4.29 × 10^−17^	39	500
23	GO:0040012	Anatomical structure morphogenesis	1.44 × 10^−16^	5.37 × 10^−16^	81	2165
53	GO:0048584	Anatomical structure formation involved in morphogenesis	2.37 × 10^−16^	3.55 × 10^−13^	45	883
70	GO:0032502	Circulatory system development	1.06 × 10^−9^	3.59 × 10^−12^	43	872
108	GO:0030334	Cell adhesion	4.59 × 10^−16^	2.76 × 10^−10^	41	925

## Data Availability

The datasets GSE152345 GSE137842 and GSE35959 used in this study are available at GEO DataSets https://www.ncbi.nlm.nih.gov/gds (accessed on 10 January 2022).
